# Opposing directions of stage-specific body shape change in a close relative of *C. elegans*

**DOI:** 10.1186/s40850-022-00131-y

**Published:** 2022-07-06

**Authors:** Eric W. Hammerschmith, Gavin C. Woodruff, Kimberly A. Moser, Erik Johnson, Patrick C. Phillips

**Affiliations:** 1 Institute of Ecology and Evolution, University of Oregon, Eugene, OR USA; 2 Princeton Neuroscience Institute, Princeton University, Princeton, NJ USA; 3 Department of Biology, University of Oklahoma, Norman, OK USA

**Keywords:** Body size, Phenotypic plasticity, Ontogenetic niche, *C. elegans*

## Abstract

**Background:**

Body size is a fundamental organismal trait. However, as body size and ecological contexts change across developmental time, evolutionary divergence may cause unexpected patterns of body size diversity among developmental stages. This may be particularly evident in polyphenic developmental stages specialized for dispersal. The dauer larva is such a stage in nematodes, and *Caenorhabditis* species disperse by traveling on invertebrate carriers. Here, we describe the morphology of a stress-resistant, dauer-like larval stage of the nematode *Caenorhabditis inopinata*, whose adults can grow to be nearly twice as long as its close relative, the model organism *C. elegans*.

**Results:**

We find that a dauer-like, stress-resistant larval stage in two isolates of *C. inopinata* is on average 13% shorter and 30% wider than the dauer larvae of *C. elegans*, despite its much longer adult stage. Additionally, many *C. inopinata* dauer-like larvae were ensheathed, a possible novelty in this lineage reminiscent of the infective juveniles of parasitic nematodes. Variation in dauer-like larva formation frequency among twenty-four wild isolates of *C. inopinata* was also observed, although frequencies were low across all isolates (< 2%), with many isolates unable to produce dauer-like larvae under conventional laboratory conditions.

**Conclusion:**

Most *Caenorhabditis* species thrive on rotting plants and disperse on snails, slugs, or isopods (among others) whereas *C. inopinata* is ecologically divergent and thrives in fresh *Ficus septica* figs and disperses on their pollinating wasps. While there is some unknown factor of the fig environment that promotes elongated body size in *C. inopinata* adults, the small size or unique life history of its fig wasp carrier may be driving the divergent morphology of its stress-resistant larval stages. Further characterization of the behavior, development, and morphology of this stage will refine connections to homologous developmental stages in other species and determine whether ecological divergence across multiple developmental stages can promote unexpected and opposing changes in body size dimensions within a single species.

**Supplementary Information:**

The online version contains supplementary material available at 10.1186/s40850-022-00131-y.

## Background

One obvious fact of the life cycles of most organisms is that they typically get larger as they develop. Indeed, most animals span at least an order of magnitude in body size within a single generation through the course of development [1, 2]. However, a frequently neglected consequence of this is that the ecological niche of an organism can change drastically within a single individual throughout development. Notable examples of such size-structured ecological niches (i.e., ontogenetic niches [3]) include animals exhibiting metamorphosis in development. Ceratophryidae frogs have larval forms that eat small crustaceans and diatoms yet grow into adult forms that eat larger arthropods, gastropods, and small vertebrates [4]. Silkworms eat mulberry leaves as larvae yet develop into short-lived, non-feeding adults specialized for reproduction [5, 6]. Such stage-specific ecology is not restricted to animals with dramatic metamorphic development. Mammals usually begin postembryonic life consuming milk before maturing into adults that consume plants, other animals, or both [1]. Many species of fish also exhibit stage-specific resource use, and largemouth bass eats planktonic crustaceans, crawfish, and cyprinid fish as it grows [3, 7].

The ontogenetic niche of an organism is not limited by resource use. Organisms often have specialized developmental stages for specific life history strategies, and such partitioning is frequently used for dispersal. Locusts have a complex polyphenism in overcrowding conditions that transform solitary morphs into gregarious morphs that constitute famine-inducing swarms [8]. Aphids also have a polyphenism specialized for dispersal where winged morphs arise in harsh conditions; dimorphic aphids also are easily distinguished by thorax size differences [9]. The seeds of plants and spores of fungi are small stress-resistant propagules that frequently harbor traits that aid in dispersal such as wings or barbs (in the case of seeds) [10, 11]. Thus life history strategies can also influence stage-specific traits including size.

As adults, the free-living bacterivorous nematodes *Caenorhabditis elegans* and *C. inopinata* live in substantially different habitats and differ substantially in size. In crowded, low food, high temperature, or otherwise stressful conditions, *C. elegans* is known to develop into a dispersal-specialized juvenile phase known as the dauer larva [12]. The dauer larva is a long-lived, desiccation-resistant, and stress-resistant dispersal stage. In its natural context, *C. elegans* dauer larvae travel to new resources on invertebrate carriers such as snails, slugs, isopods, and myriapods [13]. The dauer exhibits a stage-specific behavior in nictation, wherein the animal climbs a substrate and waves its head in the air to promote invertebrate-mediated dispersal [14]. Once it has traveled to a new rotting plant resource patch, the dauer larva will disembark and directly develop into a reproductive stage animal [13]. As the dauer larva is a specialized L3 stage, it has a distinct morphology from reproductive phase animals [12]. While a great deal is known about the *C. elegans* dauer stage, the dauer stage from *C. inopinata* has never before been described. Do the substantial differences observed in the adult species of these close relatives persist throughout this developmental stage as well?

*C. inopinata* adults grow nearly twice as long as *C. elegans*, and they also develop nearly twice as slowly as *C. elegans* [15–17]. Additionally, instead of thriving on rotting plants and dispersing on large invertebrates, *C. inopinata* lives in fresh *Ficus septica* figs and disperses on fig wasp pollinators [15, 18]. Here, we describe a stress-resistant larval stage of *C. inopinata* (which we call “dauer-like”). Rather than echoing the shape differences observed in the adults, *C. inopinata* dauer-like larvae are shorter and fatter than *C. elegans* dauers. In addition, *C. inopinata* dauer-like larvae also exhibit a novelty in ensheathment that resembles the infective larvae of parasitic nematodes. This suggests that the evolution of body shape may not be in the same direction across all developmental stages, and stage-specific ecological contexts could potentially drive opposing directions of morphological change.

## Results

**Fig. 1:**
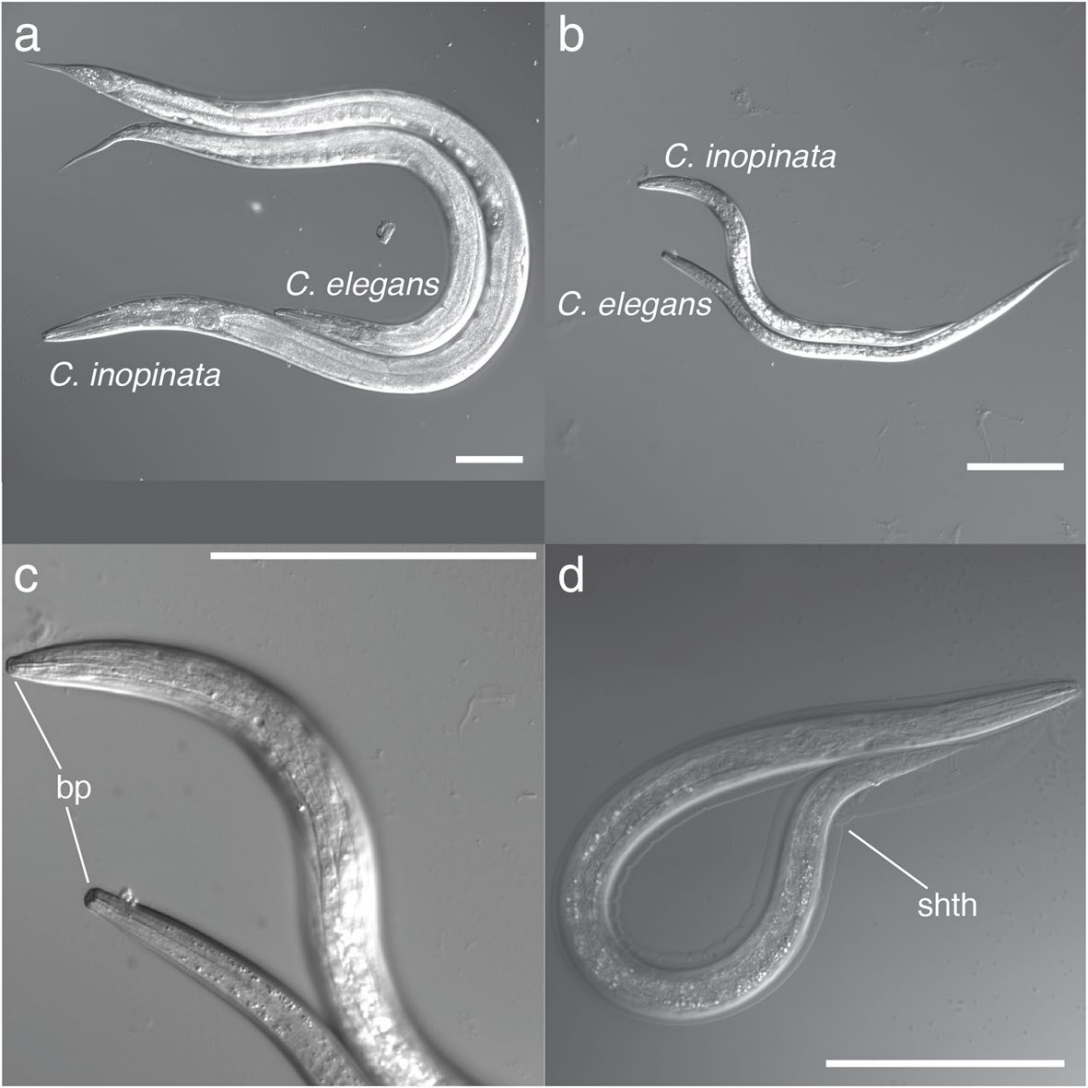
The *C. inopinata* dauer-like larva. **a***C. elegans* adult hermaphrodite with *C. inopinata* adult female. **b***C. elegans* dauer and *C. inopinata* dauer-like larvae. **c** Enlargement of panel (**b**) showing the buccal plug. Bp, buccal plug. **d** An ensheathed *C. inopinata* dauer-like larva. Shth, sheath. All scale bars represent 100 μm

*C. inopinata* adults are much longer (and marginally wider) than those of *C. elegans* (Fig. 1A [15, 16];). However, as *C. inopinata* disperses on fig wasp vectors that are millimeters long, we were curious about the extent of body size change in its dispersal dauer larva. In *C. elegans*, dauers are typically isolated via SDS exposure [19], which kills non-dauer stages that can feed and lack a buccal plug. This approach has also been shown to isolate dauer larvae in *C. briggsae* [20]. We also successfully used this approach to isolate and characterize the morphology of a stress-resistant dauer-like stage of *C. inopinata* (Fig. 1B-D). The vast majority of *C. inopinata* animals that survived SDS exposure had a buccal plug (Fig. 1C; 98%, 45/46 worms). A notable fraction of *C. inopinata* dauer-like larvae retained their cuticle from the previous molt (46%, 21/46 worms). Reminiscent of the ensheathed infective larvae of parasitic nematodes [21, 22], this was never observed in *C. elegans* dauers (*n* = 47 worms). Additionally, *C. inopinata* dauer-like larvae revealed pharyngeal constriction compared to conspecific L3 larvae (Supplemental Fig. 1; Isthmus of pharynx width 50% thinner on average, Wilcoxon rank sum test adjusted *p* = 1.1 × 10^− 20^; Pharynx fraction of total width 23% thinner on average, Wilcoxon rank sum test adjusted *p* = 4.9 × 10^− 11^). The *C. inopinata* dauer-like stage is then stress-resistant and maintains many of the hallmarks of diapause, dispersal nematodes.

**Fig. 2:**
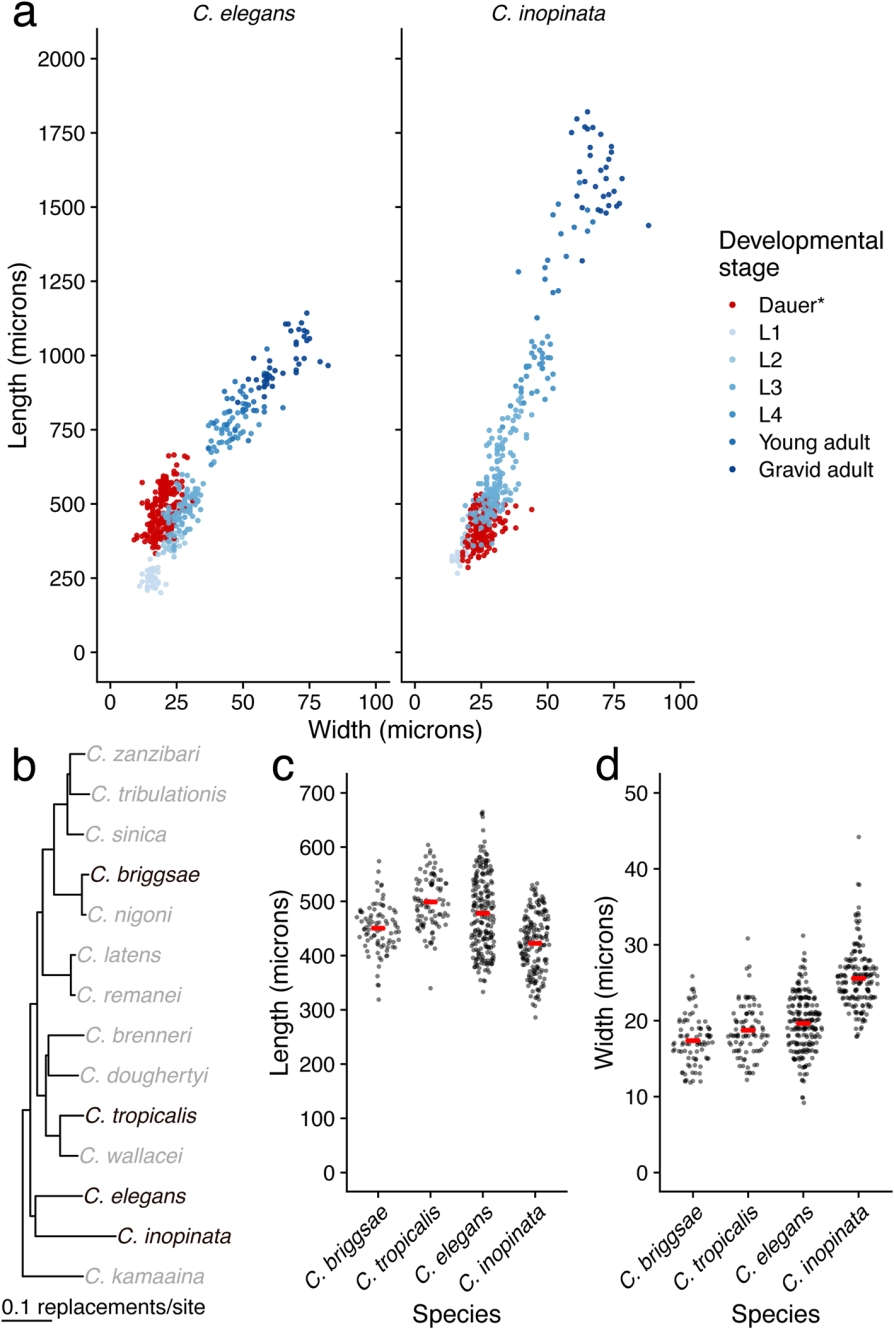
Quantification of dauer and dauer-like size. **a** Scatterplots revealing the length and width of various developmental stages. Data for non-dauer stages are from [16]. Left panel, *C. elegans*; Right panel, *C. inopinata*. Non-dauer stages N_worms_ = 16–138; *C. elegans* dauers, N_worms_ = 214; *C. inopinata* dauers, N_worms_ = 174. *Dauer-like in the case of *C. inopinata*. **b** Bayesian phylogeny of nominal *Caenorhabditis* species of the *Elegans* group from [23]. Species in black are those used for this study. **c-d** Sina plots (strip charts with points taking the contours of a violin plot) illustrating the distributions of dauer (or dauer-like in the case of *C. inopinata*) length (**c**) and width (**d**), of four *Caenorhabditis* species. Each dot represents the observation of one worm. Red bars represent averages. *C. elegans*, N_worms_ = 214; *C. inopinata*, N_worms_ = 174; *C. briggsae*, N_worms_ = 85; *C. tropicalis*, N_worms_ = 93

*C. inopinata* dauer-like larvae were shorter in length than *C. elegans* dauers (Fig. 1-2; 13% shorter on average; Wilcoxon rank sum test adjusted *p* = 7.8 × 10^− 13^; all body size summary statistics, effect sizes, and hypothesis test statistics can be found in Supplemental Table 1 sheets 1–8; Supplemental Figs. 2–3). This shorter length was observed across three independent series of measurements, albeit with different effect sizes (Supplemental Fig. 4; Supplemental Table 1 sheets 9–14; EWH 2017: 6% shorter on average, Wilcoxon rank sum test adjusted *p* = 0.01; GCW 2019: 16% shorter on average, Wilcoxon rank sum test adjusted *p* = 1.0 × 10^− 12^; KAM 2022: 17% shorter on average, Wilcoxon rank sum test adjusted *p* = 1.2 × 10^− 10^). This is surprising because all other developmental stages of *C. inopinata* were longer than comparable stages of *C. elegans* (Supplemental Figs. 2–3; 14–64% longer on average; Wilcoxon rank sum adjusted *p* = 8.6 × 10^− 13^-0.003 [16]). Because previous observations revealed *C. inopinata* adults to have shorter tail spikes [16], it was possible that differences in tail lengths were driving differences in total dauer length among species. Indeed, tails were shorter in *C. inopinata* dauer-like larvae compared to *C. elegans* dauers (Supplemental Fig. 5; 47% shorter on average; Wilcoxon rank sum test *p* = 3.4 × 10^− 14^). However, this difference in tail length does not account for all of the length difference as *C. inopinata* dauer-like larvae were shorter than *C. elegans* dauer larvae even when tails were excluded (Supplemental Fig. 5; 14% shorter on average; Wilcoxon rank sum test *p* = 3.4 × 10^− 14^). Conversely, *C. inopinata* dauer-like larvae were wider than *C. elegans* dauers (Figs. 1-2; 30% wider on average; Supplemental Figs. 3, 6; Wilcoxon rank sum test adjusted *p* = 1.7 × 10^− 35^). This increased width was observed across three independent series of measurements, albeit with different effect sizes (and an adjusted *p*-value > 0.05 in one case; Supplemental Fig. 4; Supplemental Table 1 sheets 10–14; EWH 2017: 51% wider on average, Wilcoxon rank sum test adjusted *p* = 2.3 × 10^− 29^; GCW 2019: 17% wider on average, Wilcoxon rank sum test adjusted *p* = 4.3 × 10^− 8^; KAM 2022: 5% wider on average, Wilcoxon rank sum test adjusted *p* = 0.067). These width measures do not include the sheath (an additional cuticle retained from the previous molt), as this would inflate width estimates in ensheathed *C. inopinata* animals. Regardless, the increased width of the *C. inopinata* dauer-like larva is also unexpected because previous observations of non-dauer developmental stages reveal much smaller or negligible differences in width of reproductive stages (Supplemental Figs. 3, 6; 2–8% fatter on average while the L2 is 3% thinner on average; Wilcoxon rank sum adjusted *p* = 4.7 × 10^− 4^-0.33 [16];). Thus, the length difference between *C. inopinata* and *C. elegans* increases across developmental time while the width difference remains negligible throughout development between *C. elegans* and *C. inopinata* individuals of the same non-dauer stage ([16]; Supplemental Figs. 1–2, 4). As a consequence, the linear relationship between width and length across reproductive developmental stages is steeper in *C. inopinata* than in *C. elegans* (Supplemental Fig. 7; *C. inopinata*: β_1_ = 24.4, *r*^*2*^ = 0.91, *p* = 3.4 × 10^− 135^; *C. elegans* β_1_ = 14.3, *r*^*2*^ = 0.93, *p* = 1.2 × 10^− 159^; linear model species interaction *p* = 1.6 × 10^− 63^). As *C. inopinata* dauer-like larvae are shorter and fatter than *C. elegans* dauers, they then appear to occupy different regions of morphological space relative to their respective non-dauer stages (Supplemental Fig. 7).

To further explore the hypothesis that *C. inopinata* dauer-like larvae occupy a unique position in morphological space, we also measured the dauer larvae of *C. briggsae* and *C. tropicalis*, species that are phylogenetically positioned in the clade sister to the *C. inopinata*-*C. elegans* clade ([23]; Fig. 2B). Body size is impacted by species, developmental stage, and an interaction between species and developmental stage (length-width Euclidean distances; PERMANOVA *p* = 0.001 for all terms). Pairwise comparisons reveal *C. inopinata* dauer-like larvae to occupy different regions of morphospace compared to the dauer larvae of the other species (post-hoc PERMANOVA tests; FDR-adjusted *p* = 0.001 for comparisons between the three other species; Supplemental Table 1 Sheet 15). Additionally, among all pairwise comparisons of species-stage groups, the *C. inopinata* dauer larva occupies different regions of morphospace (post-hoc PERMANOVA tests; adjusted *p* = 0.001) with the exception of the *C. inopinata* L2 larva (adjusted *p* = 0.26). *k*-means clustering likewise classifies *C. inopinata* dauers in clusters distinct from those of *C. elegans*, *C. briggsae*, and *C. tropicalis* dauers (Supplemental Figs. 8–9; Supplemental Table 1 Sheet 16; chi-square adjusted *p* = 6.8 × 10^− 48^-1.8 × 10^− 32^). In this framework, the *C. inopinata* dauer harbors distinct cluster classification from all other species-stage groups (chi-square adjusted *p* < 0.05).

Linear discriminant analysis separates *C. inopinata* dauer-like larvae from those of other species (Supplemental Fig. 10), and the discriminant function accurately classifies 81% of *C. inopinata* dauer-like larvae (29/36 animals in test set). However, the primary discriminant axis is largely driven by width (width LD1 coefficient = − 1.3; length LD1 coefficient = 0.84). Indeed, although *C. inopinata* dauer-like larvae are shorter in length than *C. elegans* dauers (Fig. 1-2, see above), *C. tropicalis* dauers (Fig. 2C; 15% shorter on average; Wilcoxon rank sum test adjusted *p* = 8.5 × 10^− 20^), and *C. briggsae* dauers (Fig. 2C; 6% shorter on average; Wilcoxon rank sum test adjusted *p* = 1.8 × 10^− 4^), these differences are small (6–15% shorter in length). Conversely, *C. inopinata* dauer-like larvae are much larger in width than the dauer larvae of all the other species (Fig. 2D; 30–47% wider on average; Wilcoxon rank sum test adjusted *p* = 1.7 × 10^− 35^-1.6 × 10^− 28^). Thus, while *C. inopinata* dauer-like larvae occupy a distinct region of morphospace compared to the dauer larvae of other species, this is largely driven by increased width. This is in stark contrast to *C. inopinata* adults, who are dramatically elongated compared to their close relatives and harbor negligible differences in width ([16]; Fig. 1-2; Supplemental Fig. 2–3, 6).

**Fig. 3:**
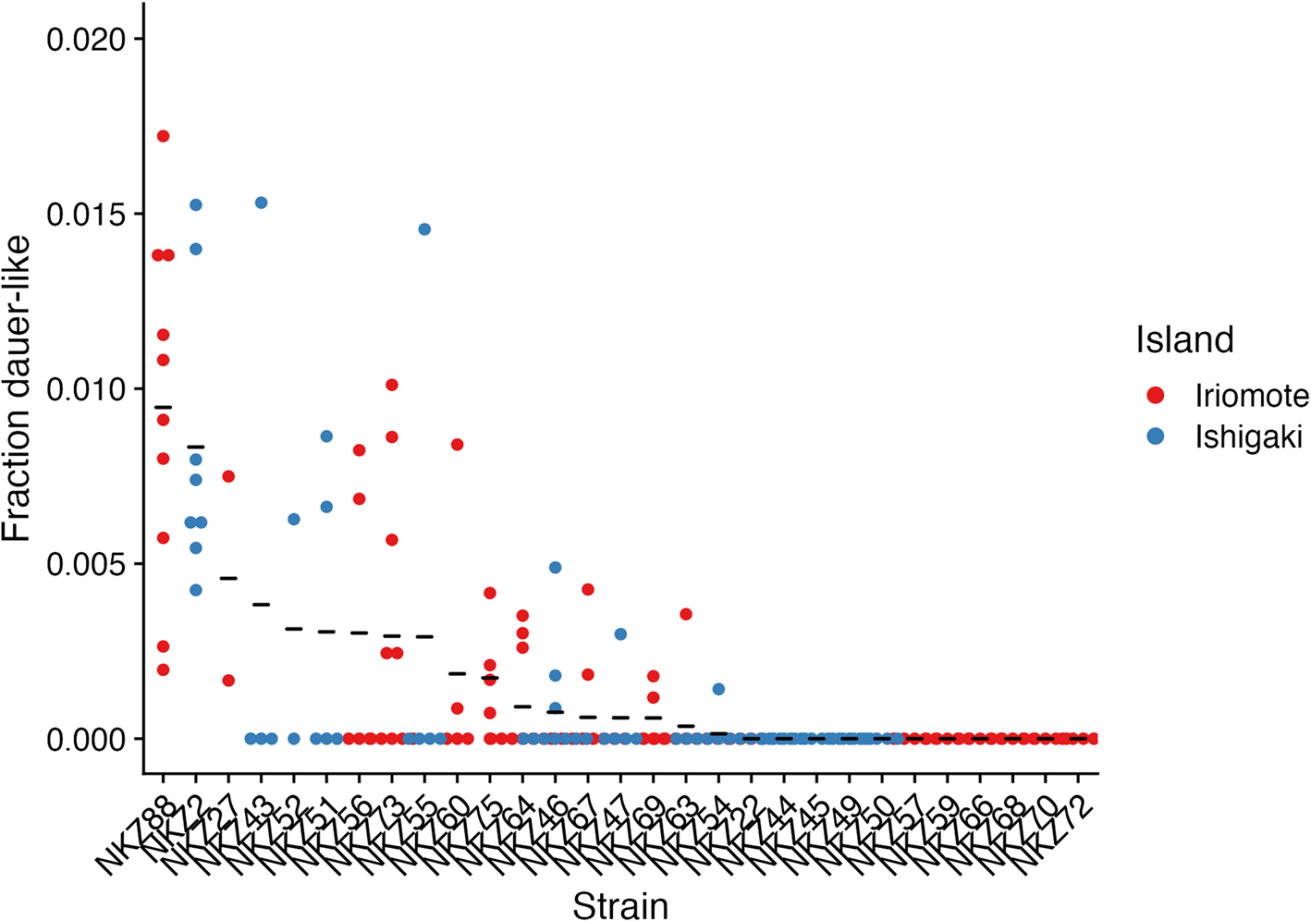
Variation in dauer-like larva formation frequency in *C. inopinata.* Twenty-five *C. inopinata* strains are shown on the x-axis; all are wild isolates with the exception of NKZ43, which is a twenty-generation inbred line derived from NKZ2. Each point represents one observation of dauer-like larva formation frequency, the fraction of a starved population of animals surviving SDS treatment (N observations per strain = 2–10; N worms per observation = 17–1557, average = 450, median = 397). Black horizontal lines represent the average. Strains are colored by Okinawan island of origin (Supplemental_tables.xls Sheet 17)

Anecdotally, it was clear that we were recovering low numbers of *C. inopinata* dauer-like larvae in our SDS treatments. As *C. inopinata* exhibits higher fitness at elevated temperatures compared to *C. elegans* [17], we reasoned higher temperatures would be needed to promote dauer formation in this species. However, dauer-like larva frequency in starved cultures at 25 °C did not differ from those raised at 30 °C (Supplemental Fig. 11; Kruskal-Wallis rank sum test chi-square = 0.015; *p* = 0.90). At 32 °C, *C. inopinata* was inviable (complete embryonic lethality, *N* = 3 plates). Additionally, in nature, dauer-like larva formation in *C. inopinata* is tied to fig and fig wasp developmental events [18], so fig or fig wasp components may be needed for dauer induction. We then prepared media made with commercially available dried figs and reared *C. inopinata* on them (fruit media prepared as in [24] (see methods)). *C. inopinata* did not proliferate on this fig media (*N* = 9 plates with complete sterility). We then continued isolating *C. inopinata* dauer-like larvae at 25 °C on NGM plates. As variation in dauer formation frequency has long been noted in *C. elegans* [25–27], we attempted to isolate dauers from a number of *C. inopinata* wild strains from Okinawan *Ficus septica* figs (Fig. 3; Supplemental Fig. 12; Supplemental Table Sheet 17). As observed in *C. elegans*, there is variation in dauer-like larva formation frequency in *C. inopinata* among 25 lines (Fig. 3; range = 0–1.7%, mean = 0.18%, sd = 0.0037). However, most lines generate dauer-like larvae at a low frequency, and many lines never produced dauer-like larvae (11 lines; Fig. 3). Furthermore, there appears to be no relationship between island of origin and dauer-like larva formation frequency (Fig. 3; Wilcoxon rank sum test *p* = 0.48, W = 4063). This is in contrast to other *Caenorhabditis* species, where many lines are able to produce abundant dauers in starvation conditions (Supplemental Fig. 13, [20, 25–27]). Thus, the ecological divergence of *C. inopinata* may have impacted the dauer entry and exit decisions in this species, leading to its low propensity to promote dauer formation in laboratory conditions.

## Discussion

The nematode dauer larva is a stress-resistant, long-lived alternative developmental stage specialized for dispersal whose development is induced by adverse environments. Here, we described the broad morphological characteristics of a stress-resistant, dauer-like larval stage of *C. inopinata*. This stage harbors many characteristics that suggest it is homologous to the dauer larval stages of other *Caenorhabditis* species. Primarily, this stage is resistant to prolonged exposure to SDS. SDS exposure is routinely used to isolate *C. elegans* dauer larvae [19], and the ability of such larvae to withstand excessive subjection to a toxic detergent underscores its life history role as a stress-resistant dispersal stage. Additionally, the *C. inopinata* dauer-like larva has a buccal plug (Fig. 1C) and exhibits pharyngeal constriction (Supplemental Fig. 1). These features are thought to be related to the dauer’s metabolic inactivity and resistance to desiccation. Additionally, the *C. inopinata* dauer-like larva was often ensheathed (Fig. 1D), a trait frequently seen in the infective juvenile stages of parasitic nematodes (that are presumed to be homologous to the dauer larvae of *Caenorhabditis* species) [21]. This additional cuticular layer is also thought to aid in stress-resistance [28–30]. Taken together, these observations suggest that this dauer-like larval stage is homologous to the dauer larva of *C. elegans*. However, other nematode life stages have been shown to be particularly resistant to stress. For instance, unlike other reproductive stages, the L1 stage of *C. elegans* can be reared in the absence of food for weeks yet still be capable of reproduction [31]. Additionally, the nematodes *Ostertagia ostertagi* and *Haemonchus contortus* harbor an L4 larval arrest state [21, 32, 33]. Moreover, it is clear that myriad aspects of *C. inopinata* biology diverge from its close relatives [15–18], and it is then possible that we have described an atypical, stress-resistant, non-dauer larval stage of *C. inopinata*. There are many hallmarks of the dauer stage that we do not explore here such as: nictation behavior [34]; cuticular reorganization [35, 36]; alae number [37]; metabolism [38]; dauer entry and exit [39]; dauer pheromones [40]; and the role of insulin signaling and other genetic pathways on dauer formation [12]. Future investigations of these aspects of *C. inopinata* biology will be invaluable in definitively connecting this stress-resistant dauer-like larva to homologous developmental stages in other *Caenorhabditis* species.

Given the above caveat, what might these observations reveal about stage-specific body shape evolution? *C. inopinata* dauer-like larvae were found to be shorter and fatter than the dauer larvae of other *Caenorhabditis* species (Fig. 2; Supplemental Figs. 2–3, 6, 13). Conversely, *C. inopinata* adults have been reported to grow as much as nearly twice [16] to three times [15] longer than *C. elegans*. Thus, its comparatively shorter and fatter stress-resistant larva is seemingly incongruent with its elongated adult form. However, this observation can be clarified by situating *Caenorhabditis* dauer larvae in their ecological contexts. Most *Caenorhabditis* species are associated with rotting plants [41, 42], and the dauer larvae of *C. elegans*, *C. briggsae*, and *C. tropicalis* have been observed to disperse on a diverse array of invertebrate carriers than span ~ 10–65 mm in body length (including gastropods, myriapods, and isopods, among others; Supplemental Fig. 14 [43, 44];). *C. inopinata*, on the other hand, thrives in an entirely different ecological context, the lumen of fresh *Ficus septica* figs [15, 18].. Furthermore, instead of dispersing on a variety of invertebrate carriers, *C. inopinata* primarily travels on *Ceratosolen* pollinating fig wasps [15, 18]. Although *C. inopinata* has been observed on the parasitic fig wasp *Phylotrypesis* [15], it appears to preferentially embark on *Ceratosolen* pollinators [15, 18]. *C. inopinata* is then notable in its degree of host vector specialization. Furthermore, fig wasp vectors are up to two orders of magnitude smaller in length than the vectors of *C. elegans* and other rotting plant-associated *Caenorhabditis* species [45]. Thus, although there must be some (as yet unknown) factor of the fig environment driving increased adult body length in *C. inopinata* adults, the divergent morphology of its stress-resistant larvae could be potentially explained by its need to disperse on much smaller vectors (Supplemental Fig. 14).

Alternatively, dauer size divergence may be caused by relaxed selection following the move to the fig environment. A hallmark of the *C. elegans* dauer larva is radial constriction, which transforms the dauer into an elongated form via autophagy, cuticular reorganization, and cell compaction [46, 47]. This leads to a dispersal stage with a length:width ratio that is double that of adult stages [46]. *C. inopinata* dauer-like larvae are not elongated in this way (Supplemental Fig. 15). Indeed, fig wasps live only a few days after maturation [48], and while radial constriction may be critical for long-term survival in most *Caenorhabditis* dauer larvae, it may be dispensable in a species that disperses to a new reproductive environment in days. The loss of radial constriction (or other reduction in dauer cuticular remodeling) is also an appealing explanation considering *C. inopinata* dauer-like larvae have similar dimensions to *C. inopinata* L2 larvae (Supplemental Figs. 2, 6). The move to a new environment may have led to the loss or reduction of this trait in *C. inopinata*, either via selection or drift, resulting in morphological divergence. In *C. elegans*, at least two genes have been characterized that are important for maintaining radial constriction in dauer larvae (*cut-6* [49] and *dex-1* [36]); when these genes are perturbed, short and fat dauer larvae result. It is possible that divergence in genetic pathways connected to these genes have led to phenotypic divergence in stress-resistant *C. inopinata* larvae. Further work in developmental biology, descriptive morphology, and ecological and evolutionary genetics will be needed to disentangle these possibilities.

Variation in dauer-like larva formation frequency was also observed in *C. inopinata* (Fig. 3). In *C. elegans*, there is substantial variation in this trait [25–27], and it has a complex genetic basis, with at least thirty-six loci contributing to most of the variance in dauer formation frequency [27]. Additionally, most isolates produced small numbers of dauer-like larvae, with many producing none at all (Fig. 3). Neither starvation at elevated temperatures (30 °C, Supplemental Fig. 10) nor rearing on media that included dried *Ficus carica* figs increased dauer-like larva formation frequency in *C. inopinata*. A caveat regarding the fig media result is that these commercial figs potentially contained a preservative that impacted nematode fitness. Regardless, in the field, *C. inopinata* dauer-like larva formation is associated with fig development and the emergence of mature pollinating fig wasps [18]. *C. inopinata* has also been shown to have higher fecundity when reared on microbes isolated from *F. septica* figs [15]. Thus, it is reasonable to suspect that compounds from *F. septica* figs, *Ceratosolen* fig wasps, or their microbial communities are needed for dauer induction in *C. inopinata*. In *C. elegans*, the dauer decision is influenced by myriad environmental inputs (such as population density, food availability, and temperature [12]), and other unexplored environmental factors are also likely important for dauer formation in *C. inopinata*. Additionally, as we did not strictly quantify nematode density in our starved cultures before dauer isolation, it is possible that variation in population density in starved cultures among species contributed to low dauer formation frequencies in *C. inopinata*. Future studies will be needed to disentangle the roles of dauer entry, exit, and their interactions with figs and fig wasps in the variation of this trait. Furthermore, we observed no clear relationship between island of origin and dauer-like larva formation frequency in these lines; this is consistent with preliminary population genomic data that reveal little genetic differentiation between Okinawan island populations [50]. Regardless, future quantitative genetic studies using these isolates will prove invaluable for understanding the genetic basis of dauer formation and other quantitative traits in *C. inopinata*.

## Conclusions

Here, we showed that a stress-resistant, dauer-like larval stage in *C. inopinata* has decreased length and increased width despite its elongated adult body size compared to *C. elegans*. This is possibly due to the divergent ecological contexts of these developmental stages that are specialized for dispersal or reproduction. Fig wasps are the dispersal vector of *C. inopinata* that are smaller and shorter-lived than those used by *C. elegans*, while figs have potentially released some selective constraint that allows longer adult body sizes compared to the rotting plant environments of *C. elegans*. Future characterization of this stage will reveal the extent of flexibility in the direction and magnitude of body shape evolution in a single species.

## Methods

### Strains and maintenance

Animals were grown on modified Nematode Growth Media with *Escherichia coli* strain OP50–1 for food as in [17]. All animals were raised at 25 °C. Strains *C. inopinata* NKZ2 (also known as NK74SC [15, 16]), *C. inopinata* PX723, *C. elegans* N2, *C. elegans* PD1074 [51], *C. briggsae* HK104, and *C. tropicalis* NIC122 were used for dauer and dauer-like larvae morphology observations. For characterizing variation in dauer formation frequency, multiple strains of *C. elegans* (ED3040, CB4856, JU1088, JU775, MY16), *C. briggsae* (JU1348, ED3091, HK104), and *C. tropicalis* (NIC122, QG131) were used. Fig media was prepared following [24]. Briefly, 20 dried *F. carica* figs (Anna and Sarah™ dried black mission figs) were blended in a kitchen blender in distilled water to make 500 mL of fig slurry, which was centrifuged for 10 minutes at 8500 rpm. Supernatant was then removed and brought up to 800 mL with distilled water. The solution was then adjusted to pH 6–7. Agar was added to 3% and the media was then prepared and seeded with bacteria as above for nematode culture.

We report multiple new wild isolates of *C. inopinata* used for characterizing dauer formation variation. *C. inopinata* strains NKZ22 and NKZ27 were isolated from fresh *F. septica* figs from the island of Iriomote, Okinawa, Japan in May 2014 (Supplemental Fig. 1; supplemental_tables.xls, Sheet 17). *C. inopinata* strains NKZ44, NKZ45, NKZ46, NKZ47, NKZ49, NKZ50, NKZ51, NKZ52, NKZ54, NKZ55, NKZ56, NKZ57, NKZ59, NKZ60, NKZ63, NKZ64, NKZ66, NKZ67, NKZ68, NKZ69, NKZ70, NKZ72, NKZ73, NKZ75, and NKZ88 were isolated from fresh *F. septica* figs from the islands of Iriomote and Ishigaki, Okinawa, Japan in May 2015 (Supplemental Fig. 12; supplemental_tables.xls, Sheet 14). *C. inopinata* strains PX723 and PX724 were isolated from fresh *F. septica* figs from Taipei, Taiwan in August 2019 (Supplemental Fig. 12; supplemental_tables.xls, Sheet 17). Wild isolates were established as in [16]. Briefly, figs were placed in a petri dish filled with water or M9 buffer. Figs were then cut into four pieces; worms subsequently found in suspension were placed on to NGM plates to establish wild isolates. Morphology and association with *F. septica* strongly suggest these wild isolates are the same species as *C. inopinata*. Molecular barcoding with the ITS2 (ribosomal rDNA internal transcribed spacer-2) sequence and/or mating tests were performed in eleven of these lines, confirming their species identity (supplemental_tables.xls, Sheet 18 [52];). As mating tests have not been performed in many lines, there exists the unlikely possibility these particular lines are cryptic species distinct from *C. inopinata*. *C. inopinata* strain NKZ43 is an inbred line of *C. inopinata* made through 20 generations of sib-pair inbreeding (derived from the genome-sequenced strain NKZ35 [15]). All animals used in this study are previously-described or newly reported invertebrate nematode strains with no prior private ownership.

### Dauer isolation

Dauer larvae were isolated from 10 to 14 day old starved cultures (derived from NGM seeded with *E. coli* OP50 as described above) with high nematode density incubated at 25 °C (with the exception of some experimental groups that were incubated at 30 °C, Supplemental Fig. 11). Animals were washed off of plates in M9 buffer and then incubated in 1% SDS for a half hour. Animals were then washed four times in M9 buffer and plated. Live worms were then used for subsequent observations.

### Microscopy and measurements

Animals were mounted on agar pads and imaged on a dissecting (“EWH 2017” data) or compound (“GCW 2019” and “KAM 2022” data) microscope. Animal length and width was measured using the ImageJ software [53]; curved lines were addressed with the “segmented line tool” as in [54]). For total animal length, animals were measured from the anterior tip of the head to the posterior tip of the tail. For width, animals were measured at the apparent mid-point of the body along the anterior-posterior axis. For tail measurements (Supplemental Fig. 5), the conventional morphological definition of the nematode tail (“the portion of the body between the anus and the posterior terminus” [55]) was not used because of concerns of variation in gut length influencing tail length measures (although gut lengths are included in the raw data). Rather, as the tail spike process (a narrow spike of cuticle that extends to the tail tip) was of interest, here the tail was defined as the length of the posterior terminus to the first visible non-cuticular feature. Sheaths were not included in measures of width, as this would inflate width estimates in ensheathed *C. inopinata* dauer-like larvae. All measures of reproductive, non-dauer stages (not including L3 larvae) were retrieved from the data in [16]. All adults in this data set directly developed into mature animals and did not pass through the dauer stage. For measures of width in that data, vulva protrusions of adult females/hermaphrodites were included. In addition, in that previous data, “young adults” were defined as recently-matured adult females/hermaphrodites with no embryos in the uterus, while “gravid adults” were defined as adult females/hermaphrodites harboring embryos in the uterus [16]. For measures of pharynxes, isthmus widths were measured from the musculature wall surrounding the lumen from side to side and approximately at the center between the corpus and grinder. The pharynx fraction of total width was measured as the isthmus width divided by the total width of worm at same location the width of the isthmus was measured. Dauer, dauer-like larvae, and additional L3 larvae size dimensions were measured across three independent series of experiments with these strains: “EWH 2017” (*C. elegans* N2 dauer; *C. inopinata* NKZ2 dauer-like larvae; *C. briggsae* HK104 dauer; *C. tropicalis* NIC122 dauer); “GCW 2019” (*C. elegans* N2 dauer; *C. inopinata* PX723 dauer-like larvae); “KAM 2022” (*C. elegans* PD1074 L3 & dauer larvae; *C. inopinata* NKZ2 L3 & dauer-like larvae). “EWH 2017” data were collected across 2 months and eleven imaging days. “GCW 2019” data were collected on one imaging day. “KAM 2022” data were collected across 2 months and eight imaging days. L3 larvae were isolated after bleach synchronization based on estimates of developmental rates at 25 °C in [16]. Sample sizes across species, stage, and experiment groups can be found in Supplemental Table 1 sheets 9–10.

Dauer formation frequency was determined as follows. Cultures on individual starved plates (as above) were washed into M9. Suspensions from single plates were split in half, and one was incubated in SDS as above while the other was incubated in M9 in parallel. All worms and carcasses were plated, and all live and dead worms were counted. Moving animals or animals with a normal, sinusoidal body posture were counted as alive (i.e., as dauer larvae); unmoving animals with a stiff, straight posture were scored as dead. The dauer formation frequency for a given culture was estimated as the fraction surviving SDS times the fraction surviving in M9. This should account for non-dauer larvae death not induced by SDS.

### Statistical analysis

All statistical analyses were performed in the R language [56]. The packages *tidyverse* [57] and *reshape2* [58] were used for data manipulation. The *ape* package [59] was used to prepare a previously published phylogenetic tree [23] for visualization. Pairwise Wilcoxon rank sum tests were performed and effect sizes were estimated with the *rstatix* package [60]; these were done to compare length and width among all species-developmental stage groups. The *lsmeans* package [61] was used for comparing linear models among species; this was used to test that the slopes of the length-width relationship of reproductive stages are different among species. PERMANOVA tests were implemented with the *vegan* [62] and *pairwiseAdonis* [63] packages; these were used to test the relationship between body size (a length and width matrix) and developmental stage/species. *K*-means clustering was performed in base R with the *kmeans* function (options iter.max = 20, nstart = 25); this was used to define clusters in length-width space. BIC was used to evaluate a range of values of *k* (*k* = 1–20), and BIC was minimized when *k* = 7. As this is also the number of developmental stages included in our data, this value was used for downstream analysis. The *caret* [64] and *MASS* [65] packages were used for linear discriminant analysis; this was used to extract linear functions that maximize the separation of species among dauer larvae. Figures were generated with the *ggplot2* [66], *cowplot* [67], *lemon* [68], *ggforce* [69], *ggmap* [70], and *ggtree* [71] packages. All data and code associated with this study have been deposited on Github (https://github.com/gcwoodruff/dauer_2020).

## Supplementary Information

**Additional file 1.**

**Additional file 2.**

## Data Availability

All data and code associated with this study have been deposited on Github (https://github.com/gcwoodruff/dauer_2020).

## Abbreviations

BIC: Bayesian information criterion
FDR: False discovery rate
L1, L2, L3, and L4: First, second, third, and fourth larval stage.
LD: Linear discriminant
NGM: Nematode growth media
PERMANOVA: Permutational analysis of variance
SDS: Sodium dodecyl sulfate
