# Supplementary material for: Opposing directions of stage-specific body shape change in a close relative of C. elegans
Source: BMC Zool. 2022 Jul 6;7:38. doi: 10.1186/s40850-022-00131-y (PMC10127021; doi:10.1186/s40850-022-00131-y)
Supplement: Supplementary file 1 — Additional file 1. [file 40850_2022_131_MOESM1_ESM.pdf]

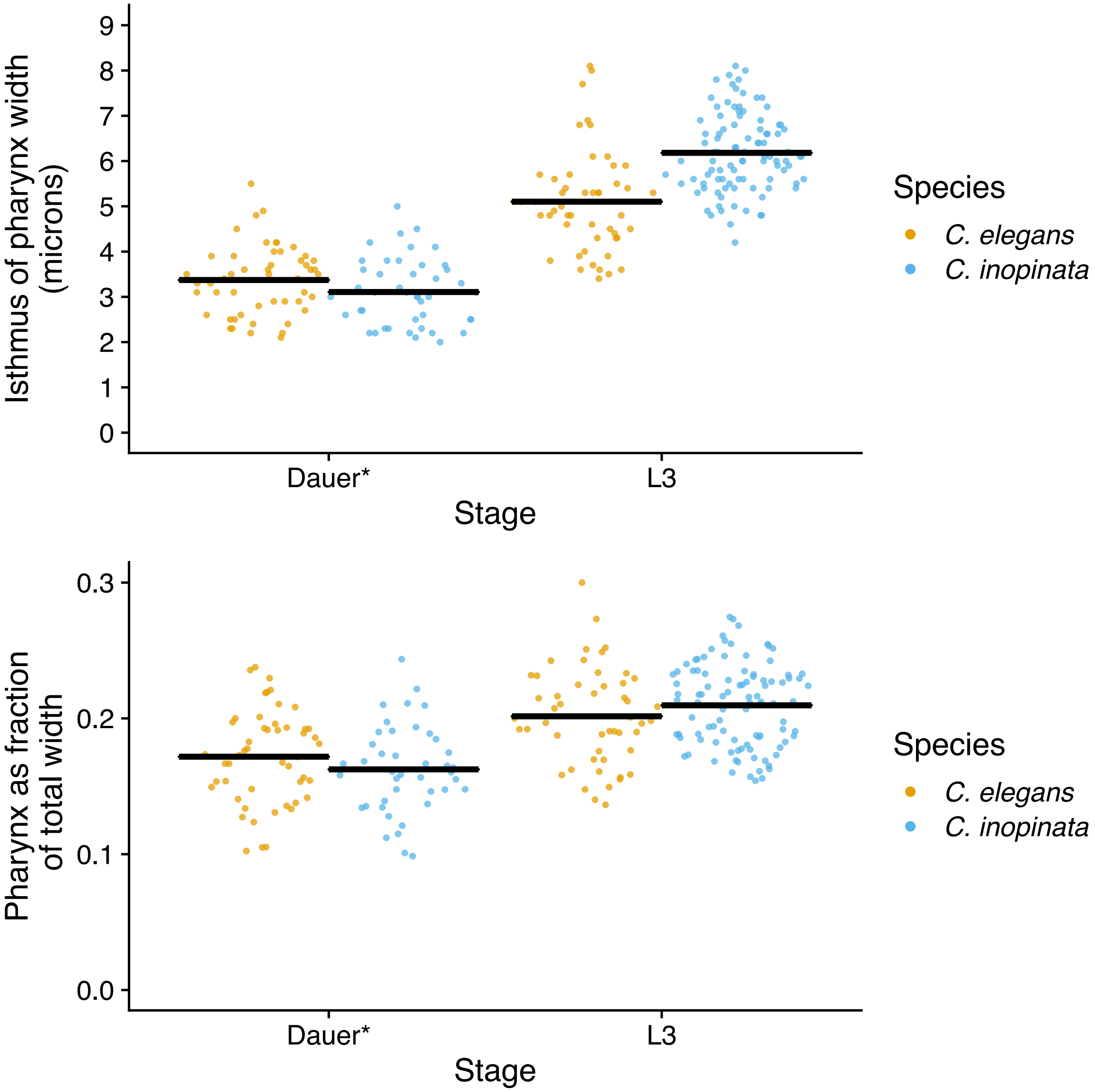

Supplemental figure 1. Pharyngeal constriction is observed in *C. elegans* PD1074 dauer larvae and *C. inopinata* NKZ2 dauer-like larvae. *Dauer-like in the case of *C. inopinata*. *C. elegans* PD1074 L3 larvae, $N_{worms} = 50$; *C. elegans* PD1074 dauer larvae, $N_{worms} = 50$; *C. inopinata* NKZ2 L3 larvae, $N_{worms} = 97$; *C. inopinata* NKZ2 dauer-like larvae, $N_{worms} = 44$.

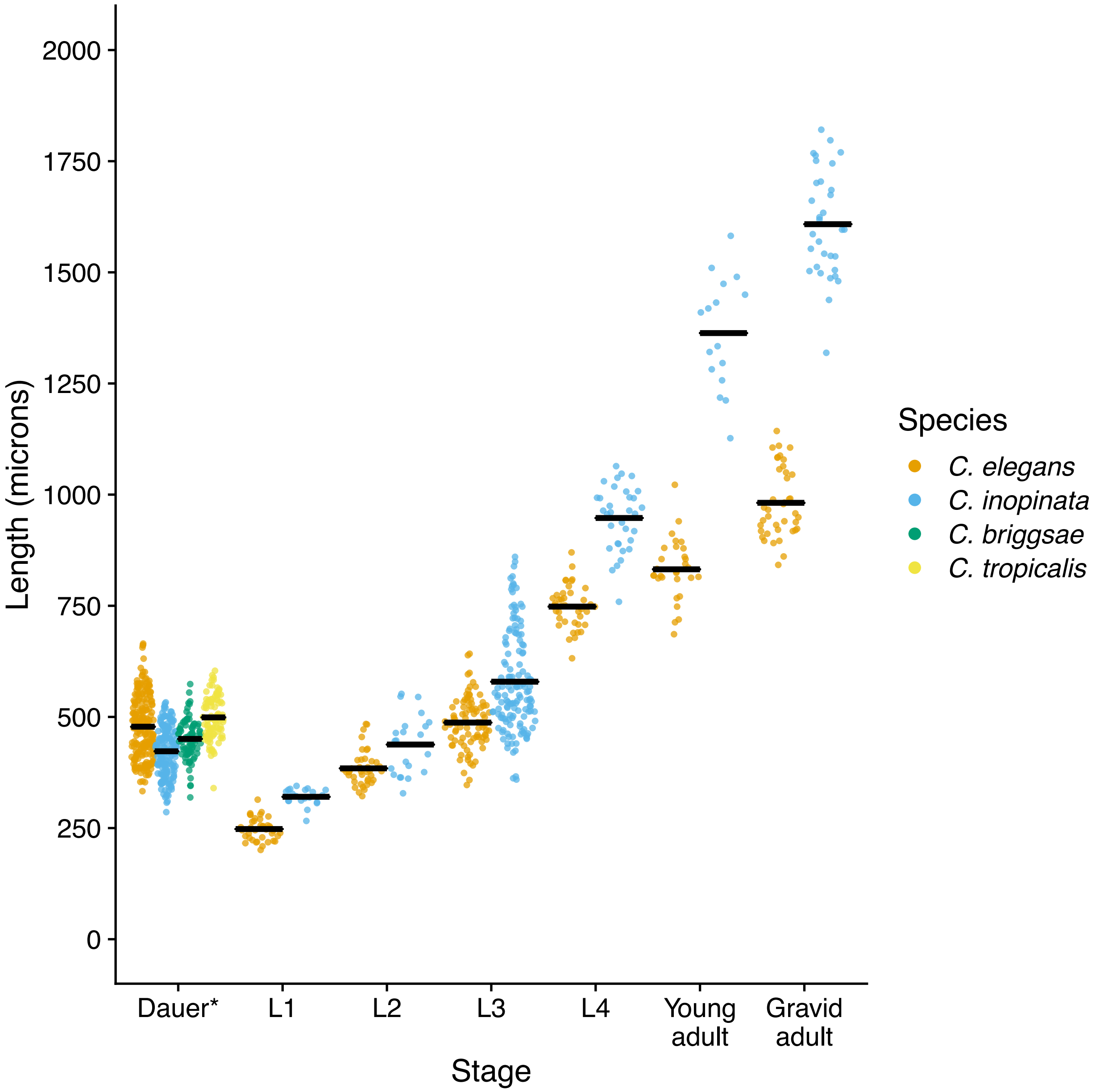

Supplemental figure 2. All *C. inopinata* developmental stages are longer than those of *C. elegans* except for the dauer/dauer-like larva. Sina plots (strip charts with points taking the contours of a violin plot) reveal length distributions among species across developmental stages. Black horizontal lines, means. *Dauer-like in the case of *C. inopinata*.

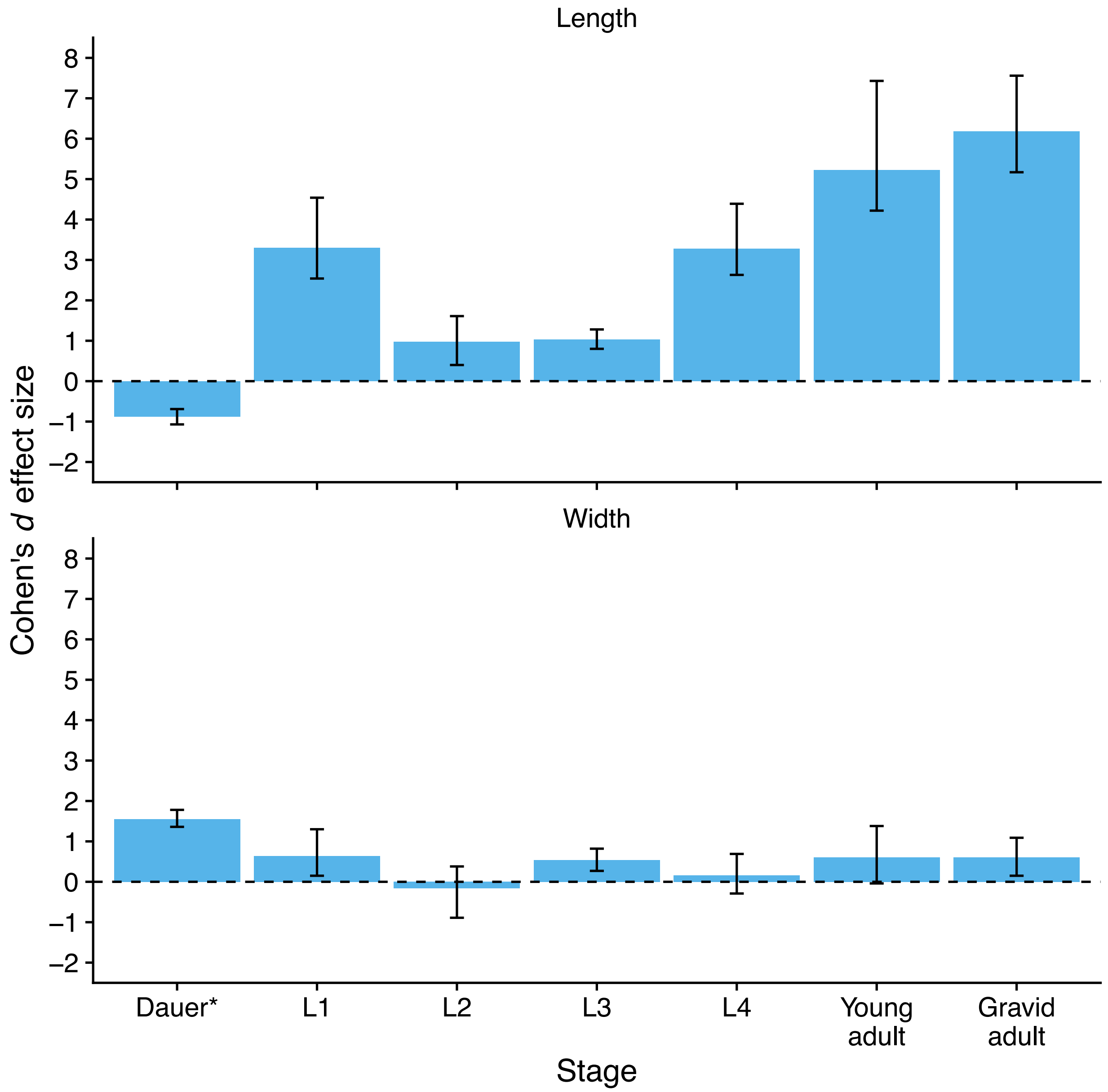

Supplemental figure 3. Effect sizes of species on length and width across developmental stages. Plotted is the Cohen's *d* effect size of species (*C. inopinata* - *C. elegans*) on size dimension (that is, $(\text{mean}_{C.\ inopinata} - \text{mean}_{C.\ elegans})/\text{sd}_{\text{pooled}}$). Here, an effect size of one notes that the average body size dimension is one pooled standard deviation higher than in *C. inopinata* than in *C. elegans*; an effect size of zero reveals on average no difference among species. Negative values reveal lengths/widths that are greater in *C. elegans* than *C. inopinata*. Error bars represent 95% confidence intervals. *Dauer-like in the case of *C. inopinata*.

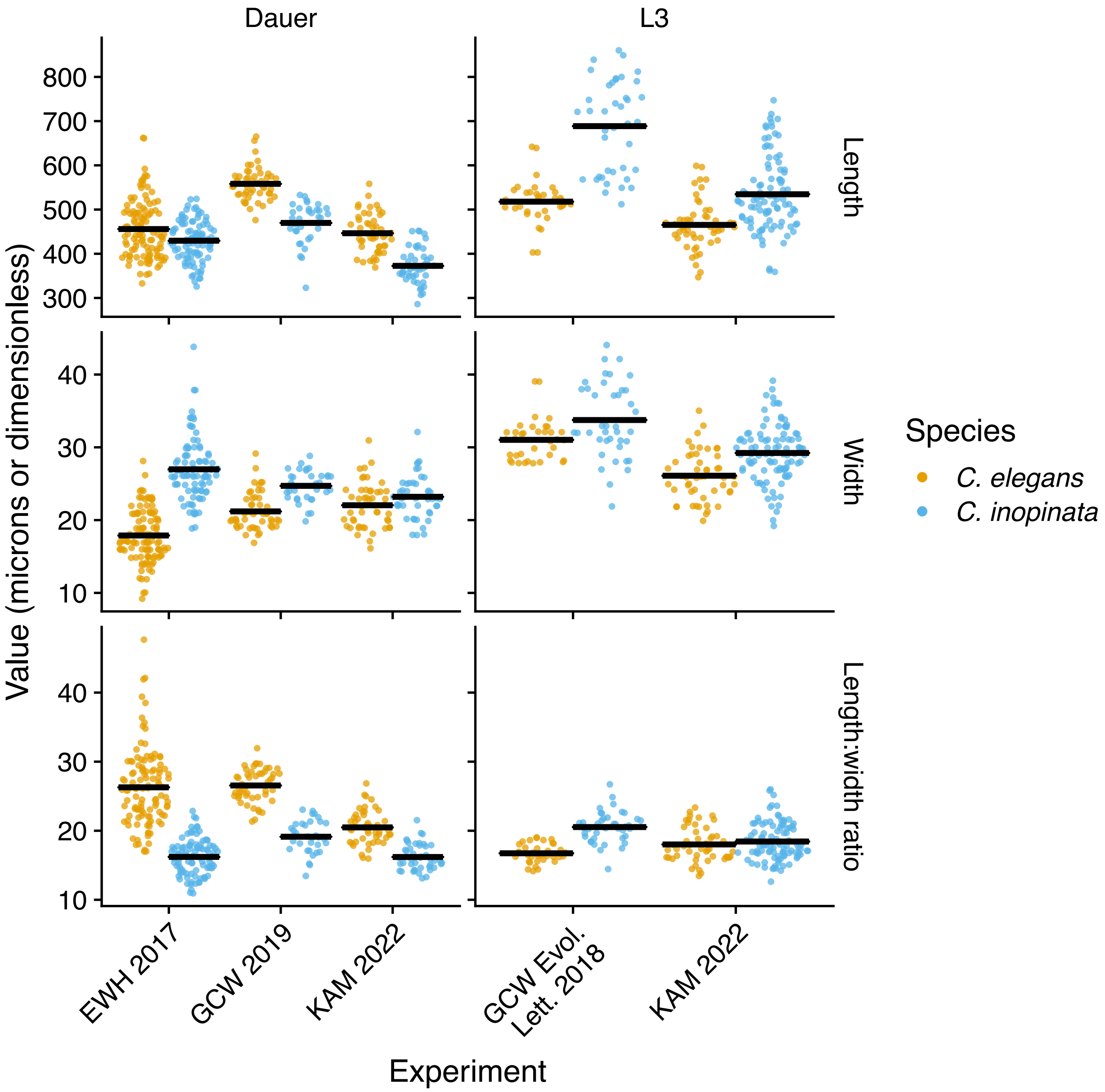

Supplemental figure 4. Trends of dauer shape divergence are reproducible across multiple experiments. *C. inopinata* dauer-like larvae sizes were estimated in three discrete experiments at different times. EWH 2017 and KAM 2022 experiments were done with *C. inopinata* NKZ2; GCW 2019 experiments were done with *C. inopinata* PX723. “GCW Evol. Lett. 2018” refers to previously published data [16] with *C. inopinata* NKZ2 & *C. elegans* N2. All other *C. elegans* observations were performed with strain N2, aside from KAM 2022, which used *C. elegans* PD1074 (a recently re-sequenced *C. elegans* N2 line [51]). *Dauer-like in the case of *C. inopinata*.

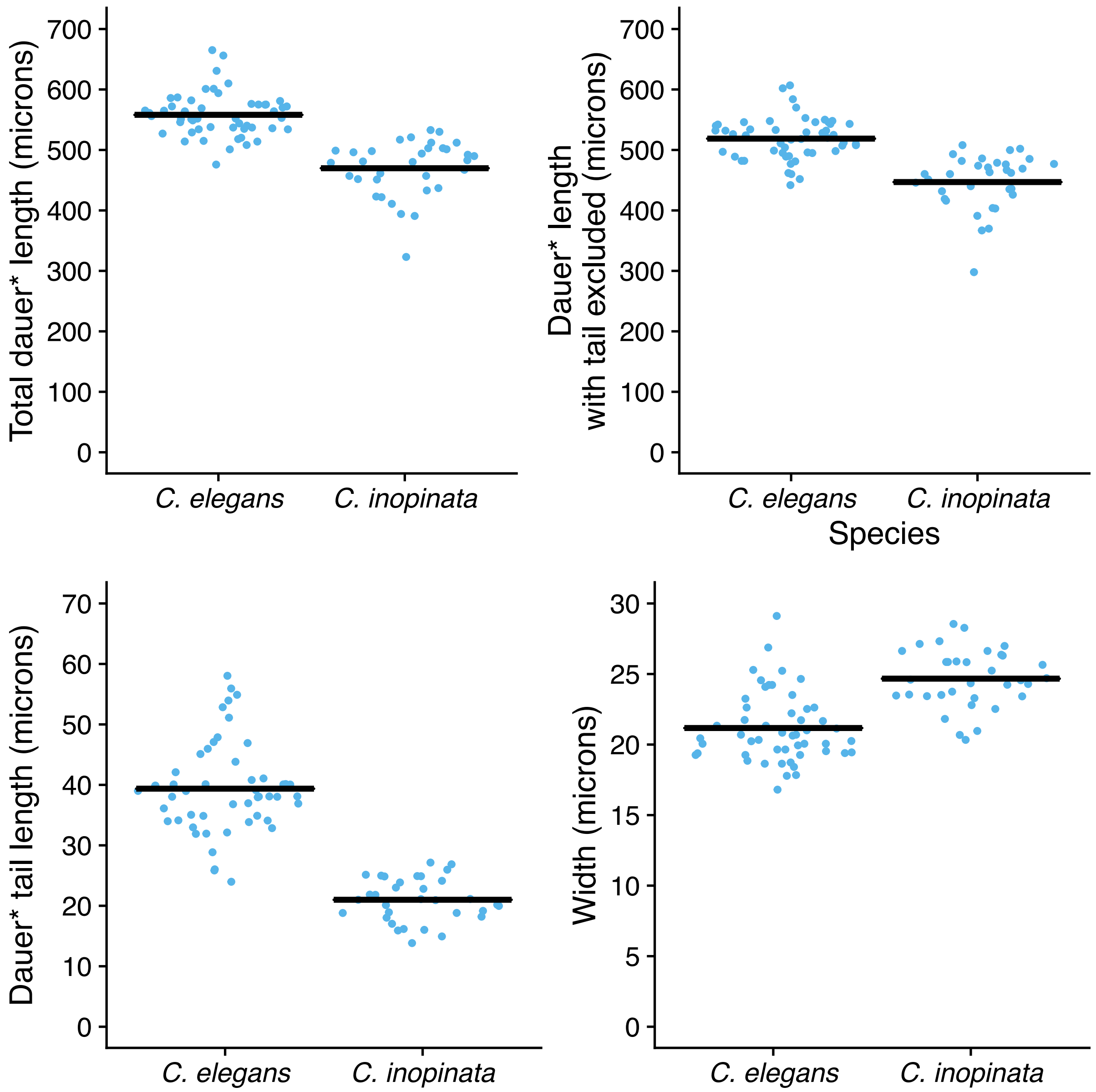

Supplemental Figure 5. Tails do not account for all of the length difference between *C. elegans* N2 dauers and *C. inopinata* PX723 dauer-like larvae. *Dauer-like in the case of *C. inopinata*.

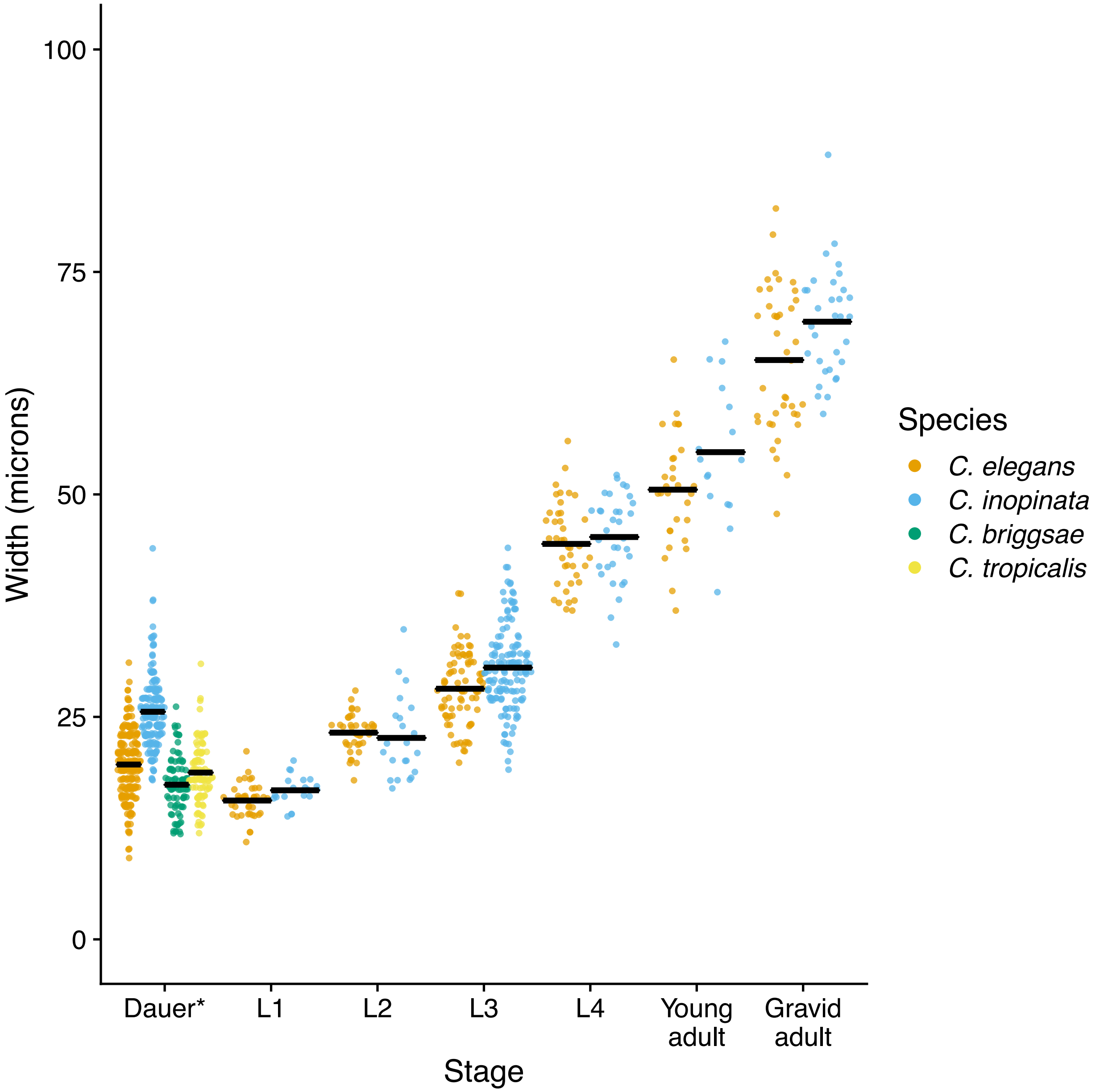

Supplemental figure 6. All *C. inopinata* developmental stages are as wide or negligibly wider than those of *C. elegans* except for the dauer larva. Sina plots (strip charts with points taking the contours of a violin plot) reveal width distributions among species across developmental stages. Black horizontal lines, means. *Dauer-like in the case of *C. inopinata*.

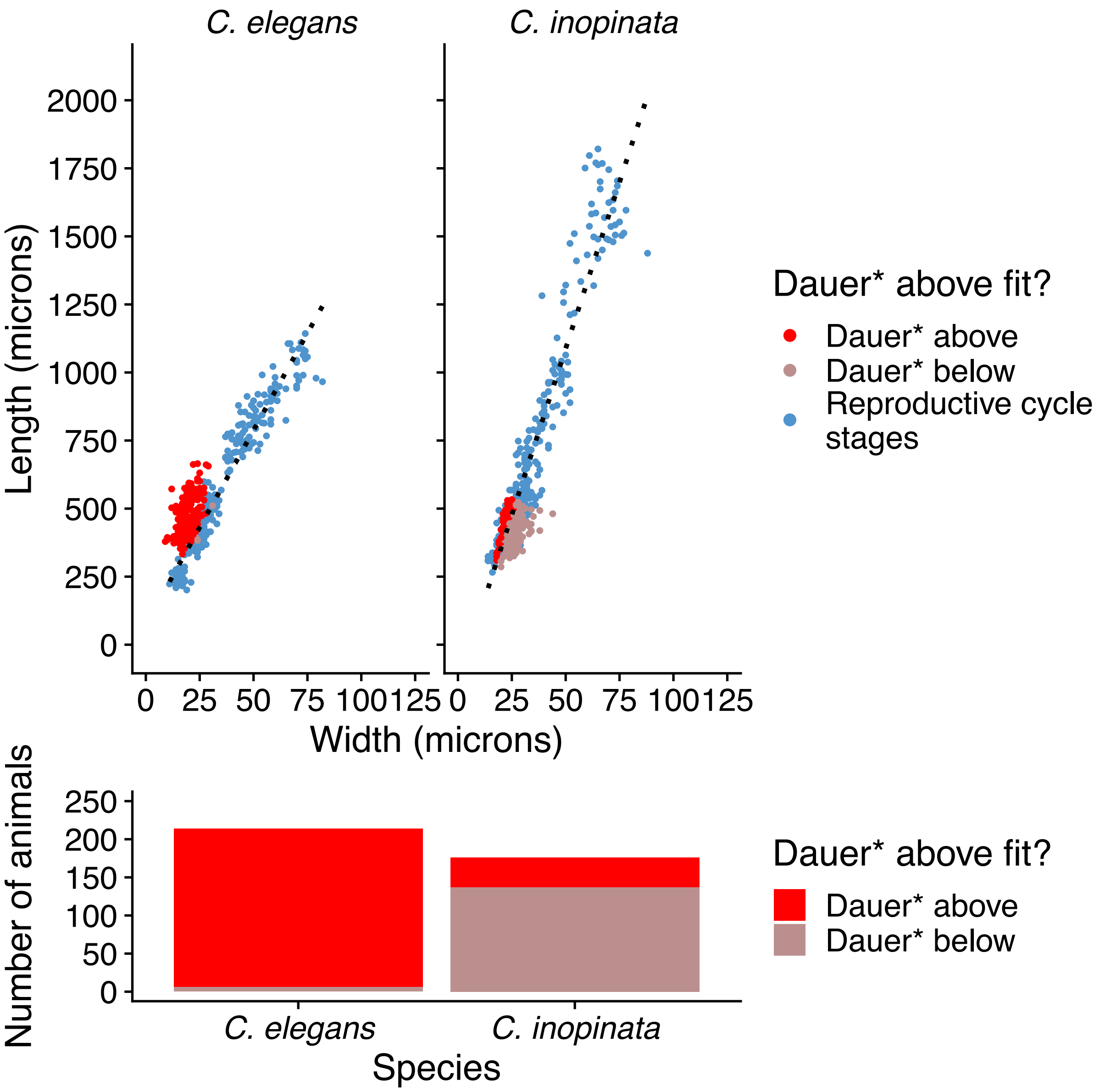

Supplemental figure 7. *C. inopinata* dauer-like larvae and *C. elegans* dauers appear to occupy different regions of morphological space relative to their respective non-dauer stages. Top panel, length-width scatterplot as in Figure 2. Blue points represent non-dauer developmental stages. Dauer larvae are colored by their relationship to a length-width linear fit for the non-dauer developmental stages to illustrate the change in dauer dimensions relative to those developmental stages. Bottom panel, a stacked bar chart revealing the difference in number of dauer larvae above the respective trendline in both species (*C. elegans* dauers above trendline: 208/214; *C. inopinata* dauers above trendline: 39/176; Chi-square = 231; $p = 3.7 \times 10^{-52}$). *Dauer-like in the case of *C. inopinata*.

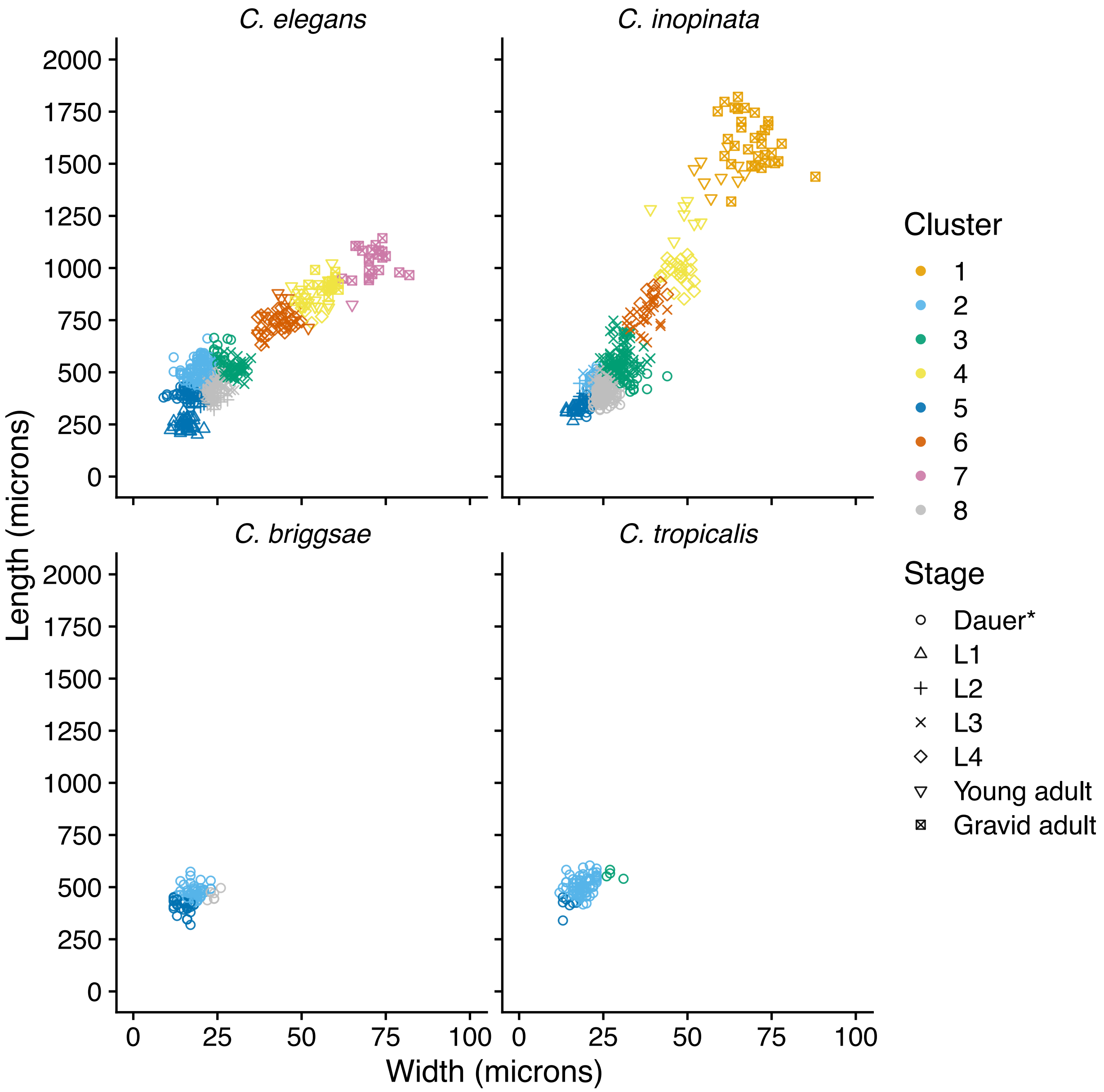

Supplemental figure 8. Distribution of animals in length-width space while colored by *k*-means clustering classification. Eight is the best value of *k* by BIC (BIC = 238). *Dauer-like in the case of *C. inopinata*.

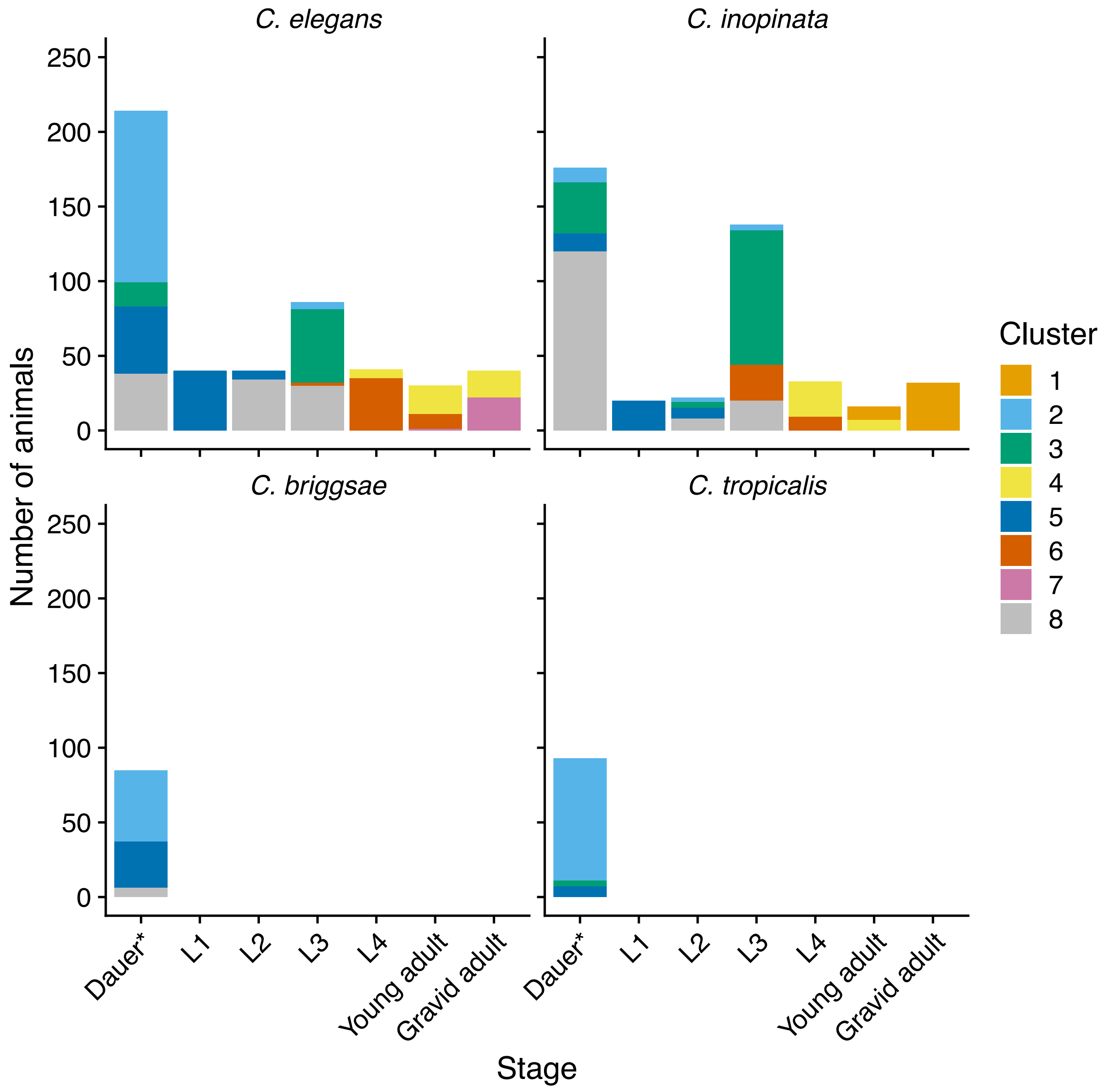

Supplemental figure 9. *C. inopinata* dauer larvae occupy different regions of morphospace compared to its close relatives following classification by *k*-means clustering. Stacked bar charts reveal the distribution of cluster classification among species and developmental stages. Same data and color scheme as in Supplemental Figure 7.

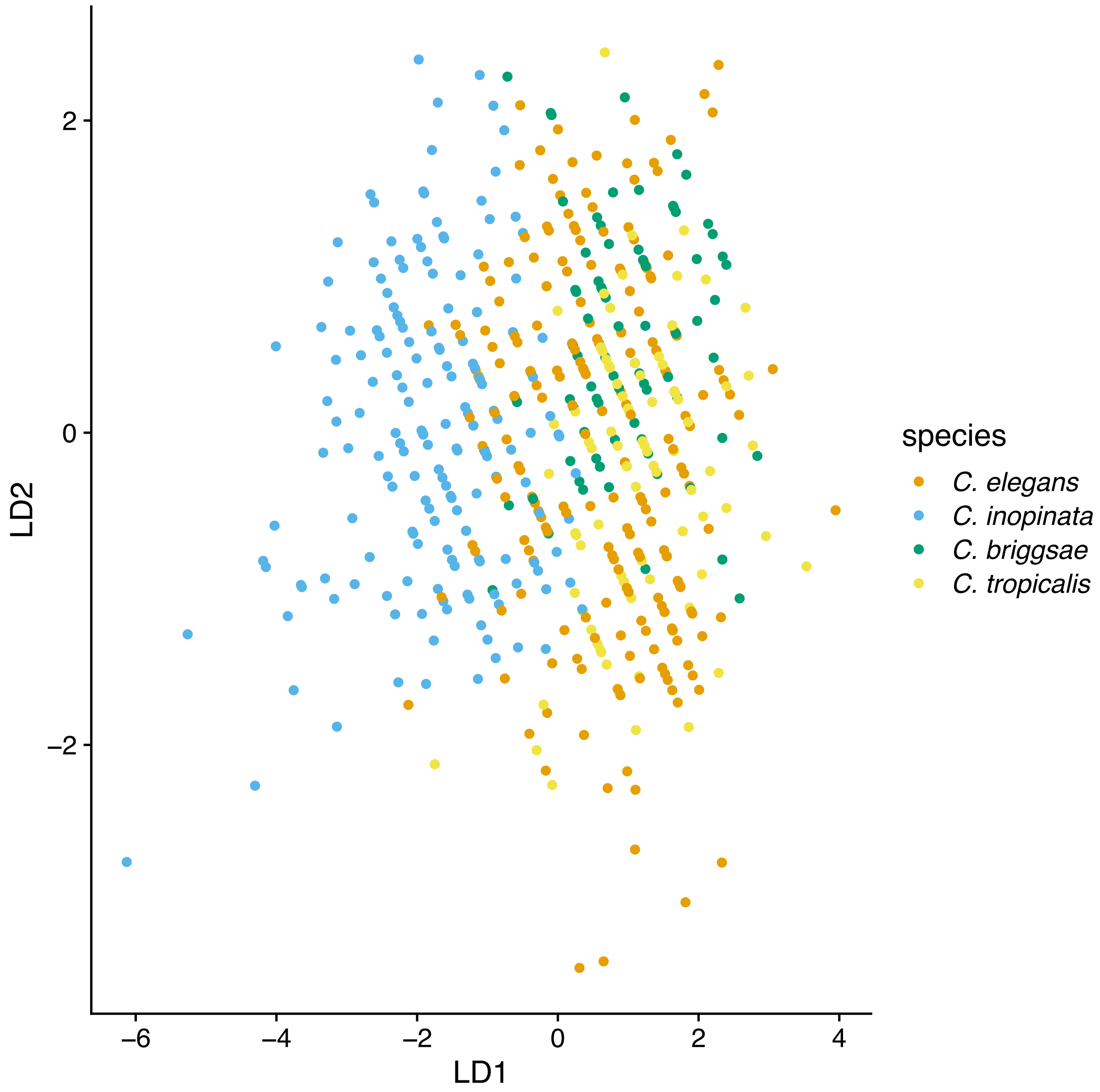

Supplemental figure 10. Linear discriminant analysis of dauer (and *C. inopinata* dauer-like) larvae body sizes. LD1 and LD2 are the component axes (linear discriminants) that maximize separation among species.

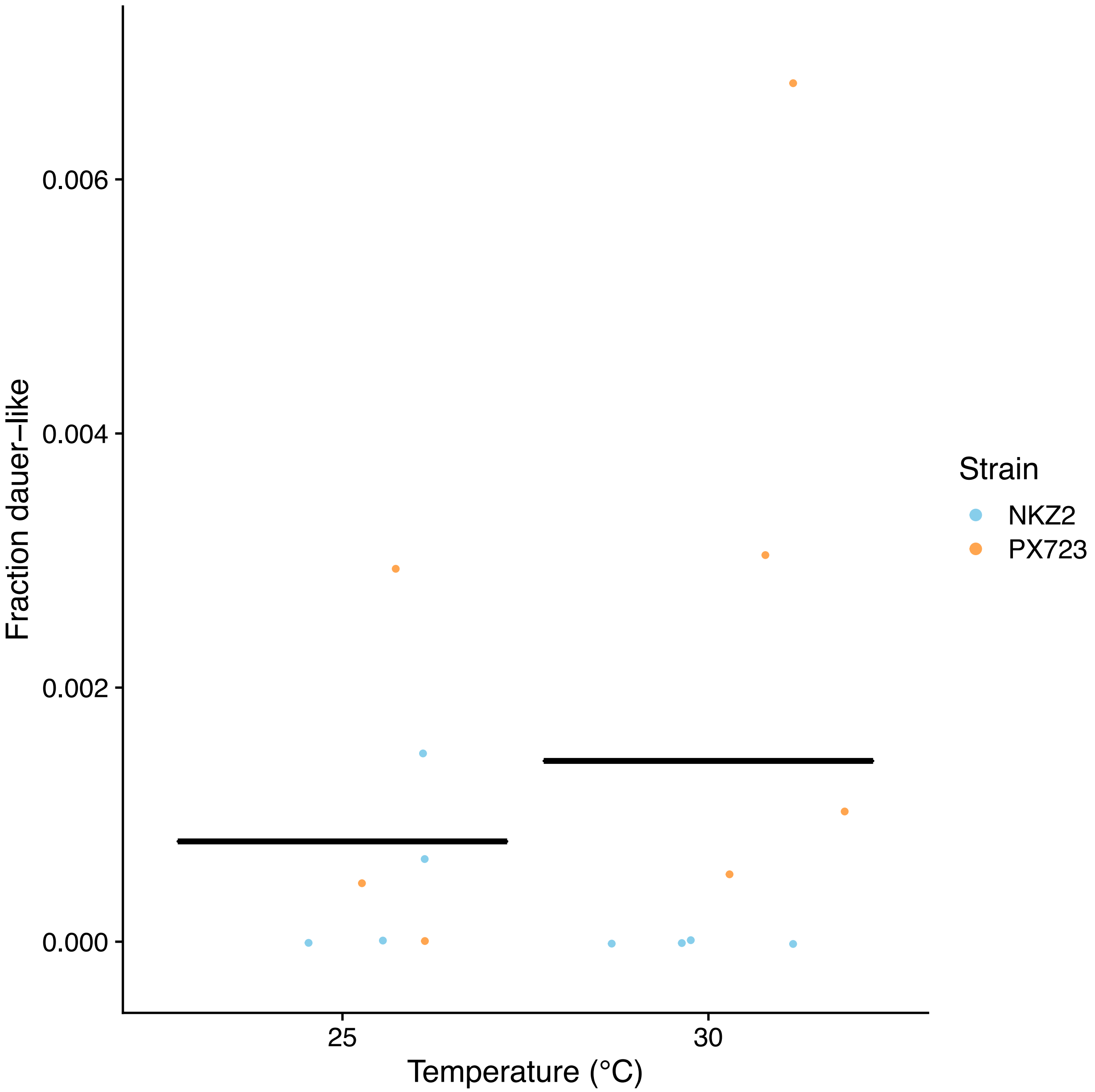

Supplemental figure 11. *C. inopinata* dauer formation frequency is unchanged at elevated temperatures. Two strains of *C. inopinata* were incubated for 10 days at 25 °C and 30 °C. Plotted are the fraction of animals surviving SDS exposure (see methods). No significant difference in dauer formation frequency between temperatures was detected (Kruskal-Wallis rank sum test chi-square = 0.015; $p = 0.90$). *C. inopinata* strain PX723 revealed a significant increase in dauer formation frequency compared to *C. inopinata* strain NKZ2 at 30 °C (Wilcoxon rank-sum test $p = 0.021$), but not after correcting for multiple tests (BH-adjusted $p = 0.13$). N total worms per plate = 130-4420.

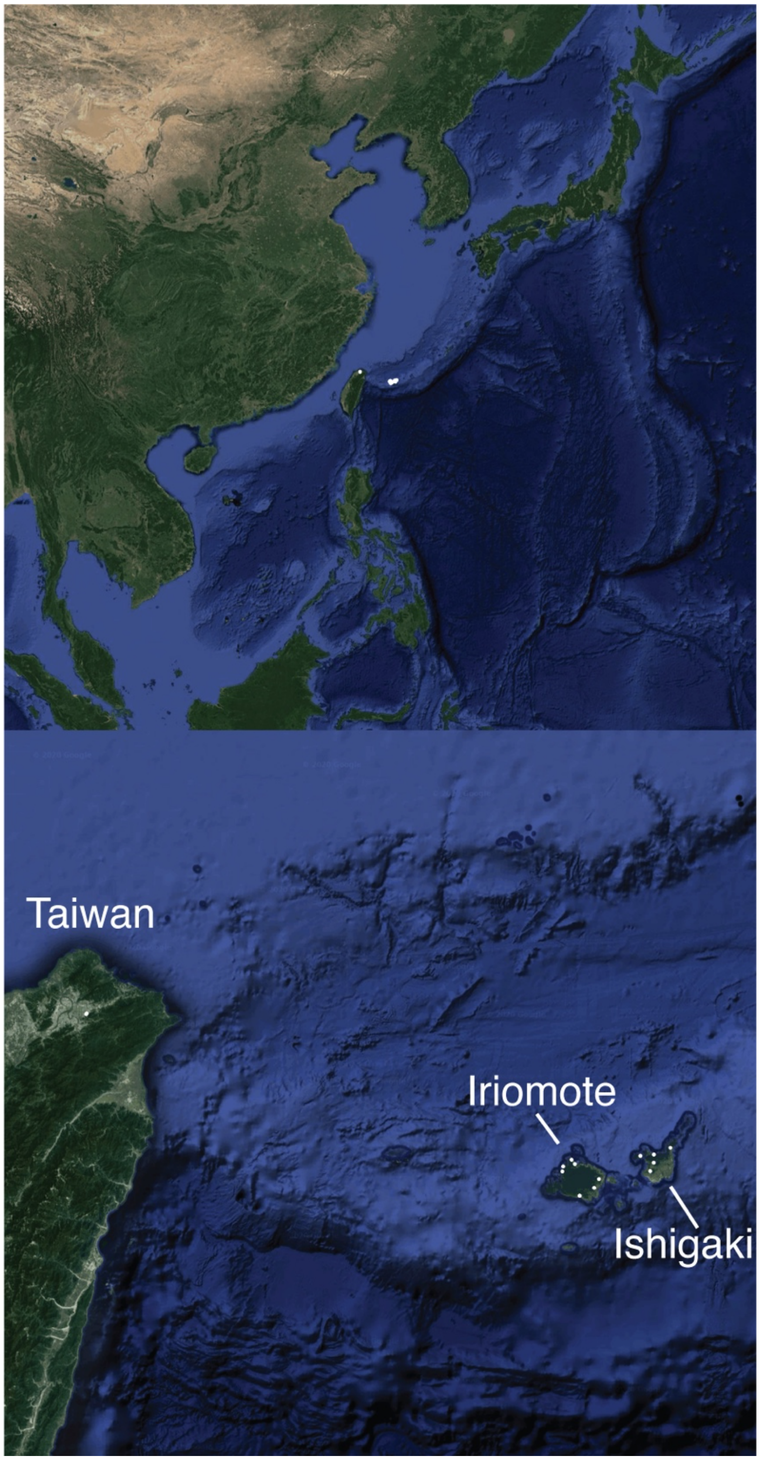

Supplemental Figure 12. Localities of *C. inopinata* strains isolated from Okinawa (islands of Iriomote and Ishigaki) and Taiwan. This map was made with an academic subscription to Google Maps in 2019 (Satellite map data ©2019 Google), and this use is allowed under their permissions policies (https://www.google.com/intl/en-GB_ALL/permissions/geoguidelines/).

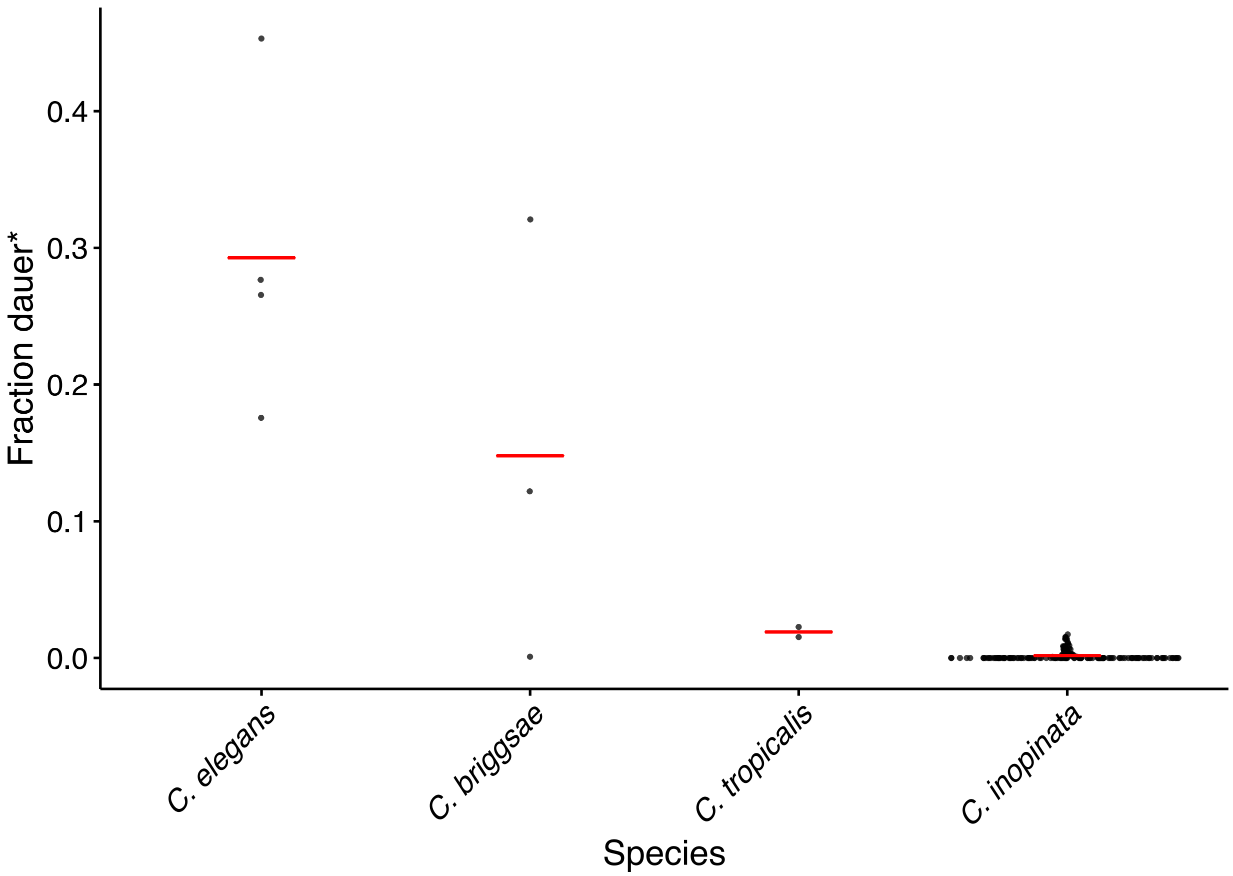

Supplemental Figure 13. *C. inopinata* produces few dauer-like larvae compared to its close relatives. Strip charts reveal the fraction of starved animals that survive SDS exposure. Red horizontal lines denote means. *Dauer-like in the case of *C. inopinata*.

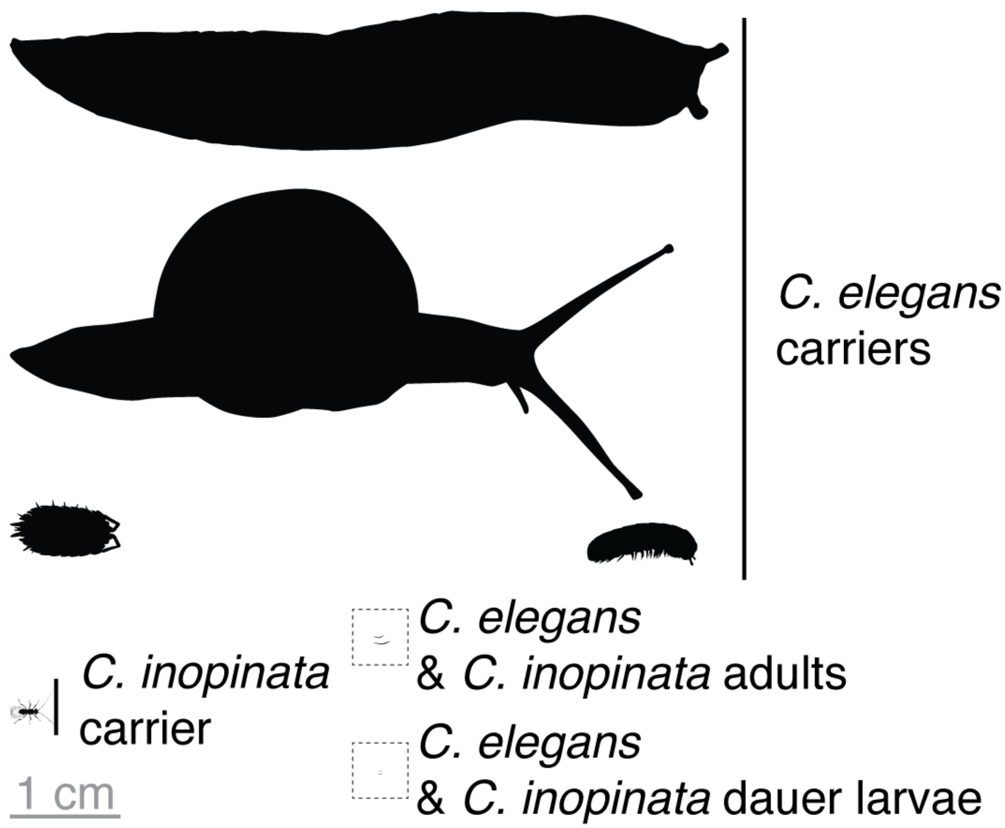

Supplemental Figure 14. Variation in the size of *Caenorhabditis* vectors. Silhouettes of invertebrates are to scale. *C. elegans* has been observed traveling on *Arion* slugs (Petersen et al. *BMC Ecology* 2015; ~65 mm long; Kozłowski et al. *Journal of Plant Protection Research* 2017); *Helix* snails (Félix and Duveau *BMC Biology* 2012; ~60 mm long; Adamo and Chase *Canadian Journal of Zoology* 1988); *Porcellio* isopods (Petersen et al. *BMC Ecology* 2015 ; ~10 mm long; Bukhari and Alikhan *Crustaceana* 1985); and *Glomeris* millipedes (Barrière and Félix *Current Biology* 2005; ~10 mm long; Beccaloni *NatSCA News* 2012). *C. inopinata* disperses on *Ceratosolen bisulcatus* fig wasps (Kanzaki et al. *Nature Communications* 2018), which are much smaller in size (~1.7 mm long; Silvieus University of Minnesota Doctoral Dissertation 2006). All silhouettes were retrieved from PhyloPic (http://phylopic.org/) and designed by Birgit Lang (isopod), Gareth Monger (gastropods), Thomas Hegna (millipede), and Kamil S. Jaron (wasp).

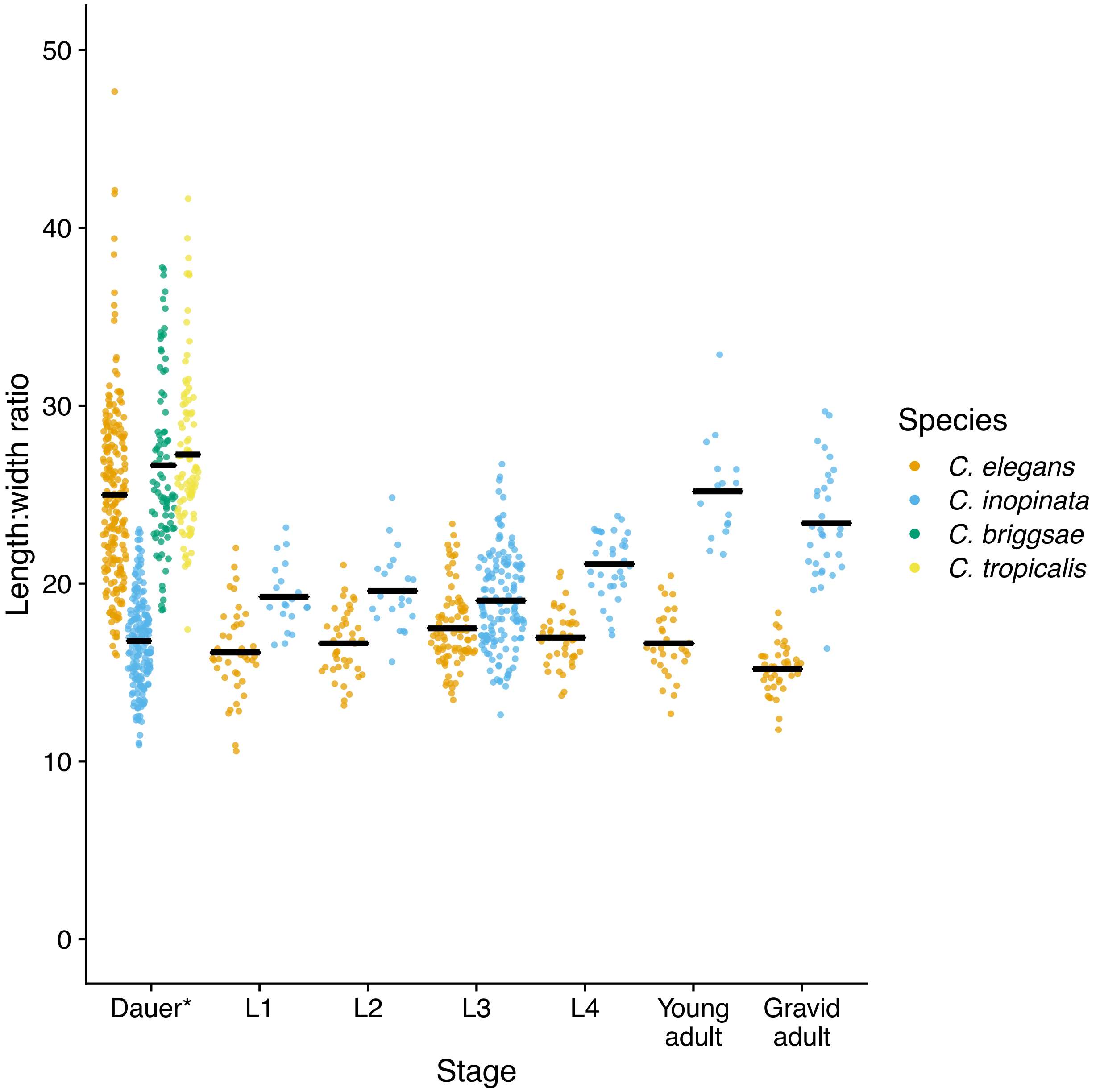

Supplemental figure 15. *C. inopinata* dauers are not elongated compared to their reproductive stages. Sina plots (strip charts with points taking the contours of a violin plot) reveal length:width ratio distributions among species across developmental stages. Black horizontal lines, means. *Dauer-like in the case of *C. inopinata*.
